# Genome-Scale Modeling of Light-Driven Reductant Partitioning and Carbon Fluxes in Diazotrophic Unicellular Cyanobacterium *Cyanothece* sp. ATCC 51142

**DOI:** 10.1371/journal.pcbi.1002460

**Published:** 2012-04-05

**Authors:** Trang T. Vu, Sergey M. Stolyar, Grigoriy E. Pinchuk, Eric A. Hill, Leo A. Kucek, Roslyn N. Brown, Mary S. Lipton, Andrei Osterman, Jim K. Fredrickson, Allan E. Konopka, Alexander S. Beliaev, Jennifer L. Reed

**Affiliations:** 1 Department of Chemical and Biological Engineering, University of Wisconsin- Madison, Madison, Wisconsin, United States of America; 2 Biological Sciences Division, Pacific Northwest National Laboratory, Richland, Washington, United States of America; 3 Burnham Institute for Medical Research, La Jolla, California, United States of America; Boston University, United States of America

## Abstract

Genome-scale metabolic models have proven useful for answering fundamental questions about metabolic capabilities of a variety of microorganisms, as well as informing their metabolic engineering. However, only a few models are available for oxygenic photosynthetic microorganisms, particularly in cyanobacteria in which photosynthetic and respiratory electron transport chains (ETC) share components. We addressed the complexity of cyanobacterial ETC by developing a genome-scale model for the diazotrophic cyanobacterium, *Cyanothece* sp. ATCC 51142. The resulting metabolic reconstruction, *i*Cce806, consists of 806 genes associated with 667 metabolic reactions and includes a detailed representation of the ETC and a biomass equation based on experimental measurements. Both computational and experimental approaches were used to investigate light-driven metabolism in *Cyanothece* sp. ATCC 51142, with a particular focus on reductant production and partitioning within the ETC. The simulation results suggest that growth and metabolic flux distributions are substantially impacted by the relative amounts of light going into the individual photosystems. When growth is limited by the flux through photosystem I, terminal respiratory oxidases are predicted to be an important mechanism for removing excess reductant. Similarly, under photosystem II flux limitation, excess electron carriers must be removed *via* cyclic electron transport. Furthermore, *in silico* calculations were in good quantitative agreement with the measured growth rates whereas predictions of reaction usage were qualitatively consistent with protein and mRNA expression data, which we used to further improve the resolution of intracellular flux values.

## Author Summary

Cyanobacteria have been promoted as platforms for biofuel production due to their useful physiological properties such as photosynthesis, relatively rapid growth rates, ability to accumulate high amounts of intracellular compounds and tolerance to extreme environments. However, development of a computational model is an important step to synthesize biochemical, physiological and regulatory understanding of photoautotrophic metabolism (either qualitatively or quantitatively) at a systems level, to make metabolic engineering of these organisms tractable. When integrated with other genome-scale data (*e.g.*, expression data), numerical simulations can provide experimentally testable predictions of carbon fluxes and reductant partitioning to different biosynthetic pathways and macromolecular synthesis. This work is the first to computationally explore the interactions between components of photosynthetic and respiratory systems in detail. *In silico* predictions obtained from model analysis provided insights into the effects of light quantity and quality upon fluxes through electron transport pathways, alternative pathways for reductant consumption and carbon metabolism. The model will not only serve as a platform to develop genome-scale metabolic models for other cyanobacteria, but also as an engineering tool for manipulation of photosynthetic microorganisms to improve biofuel production.

## Introduction

*Cyanothece* spp. are unicellular, diazotrophic cyanobacteria that temporally separate light-dependent oxygenic photosynthesis and glycogen accumulation from N_2_ fixation at night [1]. When grown under nutrient excess, *Cyanothece* sp. strain ATCC 51142 (thereafter *Cyanothece* 51142) cells can accumulate significant amounts of storage polymers including glycogen, polyphosphates, and cyanophycin [2]. The inter-thylakoid glycogen granules are significantly larger in size than those found in other cyanobacteria, which points at an unusual branching pattern and packaging of this compound. From a biotechnological perspective, this presents an intriguing theoretical possibility to accumulate substantially higher amounts of polyglucose without any significant increase in the number of granules [3]. *Cyanothece* 51142 is also of interest for bioenergy applications due to its ability to evolve large quantities of H_2_. Remarkably, H_2_ production in this organism can occur under light conditions in the presence of O_2_ and is mediated by nitrogenase [4], [5]

Sequencing of the *Cyanothece* 51142 genome [6] has enabled application of high-throughput genomic approaches to study the unique physiological and morphological features of this organism. Transcriptomic and proteomic studies have been conducted to analyze global gene expression patterns under a variety of environmental conditions and infer regulatory pathways that govern the organism's diurnal growth [7], [8]. The availability of genomic information also provides means to construct genome-scale constraint-based models of metabolism, which are powerful tools for systems-level analysis and prediction of biological systems response to environmental cues and genetic perturbations [9], [10]. Such models have been developed for a variety of biological systems [9] but only in a few studies has this approach been applied to photosynthetic microorganisms, including *Synechocystis* sp. PCC 6803 [11]–, *Rhodobacter sphaeroides* [14], and *Chlamydomonas reinhardtii* [15], [16]. However, the modeling of metabolism in oxygenic photoautotrophs is an intriguing problem due to the complexity of photosynthetic and respiratory electron transport chains, and the potential effects of two distinct photosystems upon the generation and fate of reductant and energy that drives the remainder of metabolism.

In this work, we developed the first genome-scale metabolic model of *Cyanothece* 51142 and used a combination of computation and experimental approaches to investigate how photosynthetic and respiratory fluxes affect metabolism. Discrete representation of PS II and PS I and their integration with multiple respiratory pathways enabled modeling of photon fluxes and electron flux distributions under conditions of variable light quality and intensity. The predicted changes in growth rates of *Cyanothece* 51142 in response to changes in light input were experimentally tested using a photobioreactor with controlled sources of monochromatic 630 and 680 nm light. We also carried out computational and experimental analyses of light- and nitrogen-limited chemostat growth of *Cyanothece* 51142 and used mRNA and protein expression data to constrain model-predicted flux distributions. Both *in silico* and experimental data suggest that respiratory electron transfer plays a significant role in balancing the reductant (NADPH) and ATP pools in the cells during photoautotrophic growth. This study is a first step towards a systems-level analysis of cyanobacterial metabolism, as it integrates information into a genome-scale reconstruction to understand metabolism qualitatively and quantitatively through a constraint-based analysis [9]. We also discuss strategies for improving internal flux distributions through integration of *in silico* simulations and data.

## Results

### Metabolic network reconstruction and initial model validation

**Figure 1:**
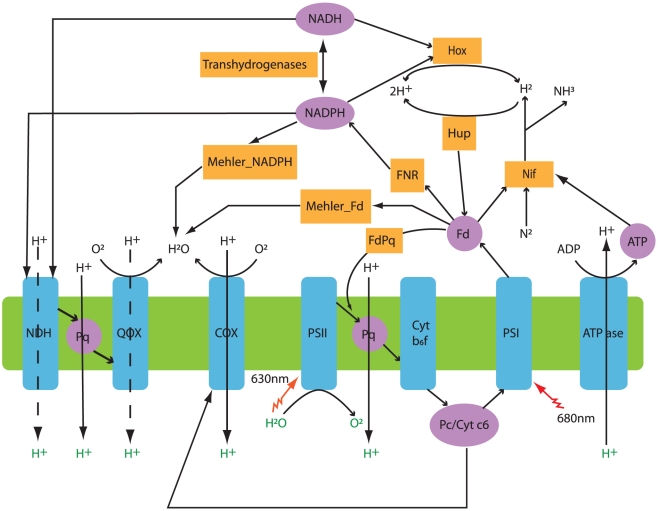
Schematic representation of the electron transport and reductant partitioning pathways in *Cyanothece* 51142. *Linear photosynthetic electron transfer*: electrons from photosystem II (PS II) to photosystem I (PS I) are transferred through plastoquinone (Pq), cytochrome *b_6_f* complex (Cyt b6f), plastocyanin (Pc) and cytochrome *c_6_* (Cyt c6). From PS I electrons can be transferred to ferredoxin (Fd) *via* ferredoxin:NADP^+^ reductase (FNR) and subsequently to generate reductant in the form of NADPH. *Cyclic photosynthetic electron transport*: electrons can flow from Fd to Pq (FdPq reaction). *Respiratory electron transfer*: includes two cytochrome oxidases (COX), two cytochrome-quinol oxidases (QOX), and two types of NADH dehydrogenases (NDH-1 and NDH-2). *Alternative sinks for reductant beyond CO_2_ fixation*: reduced Fd can be used by the nitrogenase (Nif) and by Mehler reactions to reduce O_2_. Bidirectional hydrogenase (Hox) can reversibly produce H_2_ using NAD(P)H as an electron donor, while the uptake hydrogenase (Hup) consumes H_2_ using Fd as an electron acceptor. Protons transferred across the thylakoid membrane are used by the ATPase to drive ATP synthesis.

To build a constraint-based metabolic model of *Cyanothece* 51142, a genome-scale metabolic network was reconstructed using the genome annotation and data from NCBI [6], SEED [17], KEGG [18]–[20], and CyanoBase [21], [22]. The resulting *i*Cce806 network contains 806 genes and 667 metabolic and transport reactions (see Dataset S1 and Tables S1, S2, S3 for network details). Most of the 42 reactions without genes associated with them were added to complete metabolic pathways needed for biomass production. The final reconstruction encompasses central metabolic pathways such as the Calvin-Benson cycle, the pentose phosphate pathway (PPP), reactions within the tricarboxylic acid (TCA) cycle, as well as, the complete set of anabolic pathways involved in biosynthesis of glycogen, cyanophycin, amino acids, lipids, nucleotides, vitamins, and cofactors. Pathways for glycolate synthesis (*via* ribulose-1,5-bisphosphate carboxylase/oxygenase, *i.e.*, photorespiration), glycolate conversion to serine, and glycerol catabolism are also included. Photosynthetic electron transfer associated with the thylakoid membrane is represented as a set of four separate reactions, including light capture by photosystem II (PS II) and photosystem I (PS I), electron transfer between the two photosystems, and cyclic electron transfer around PS I. Similarly, respiratory electron transfer is represented by reactions catalyzed by terminal cytochrome *c* oxidase (COX), quinol oxidases (QOX, both *bd*- and *bo*-types), NADH dehydrogenases (NDH, type 1 and 2), and succinate dehydrogenase. In addition, two reactions (NADP^+^- and ferredoxin- requiring) for flavin-dependent reduction of O_2_ (*i.e.*, Mehler reactions) were included. A simplified scheme of the photosynthetic and respiratory electron transfer reactions in *i*Cce806 is shown in Figure 1.

For initial testing, we examined the ability of the constraint-based model of *i*Cce806 to predict growth under photoautotrophic (using light and fixing CO_2_), heterotrophic (using glycerol in the dark), and photoheterotrophic (using glycerol and light) conditions with different nitrogen sources. *In silico* calculated biomass yields, which simulated carbon or light- limited growth (Figure S1), qualitatively agreed with previously reported growth data for *Cyanothece* 51142 [1], [2], [23]. Other non-growth conditions that were simulated with the model, included nitrogen fixation as occurs during the dark phase of *Cyanothece*'s ciracadian cycle [1]. In this case, the oxidation of glycogen provides reductant and ATP for nitrogenase, and we examined the model's ability to quantitatively predict the amount of nitrogen (N_2_) that could be fixed and stored in the dark, by maximizing cyanophycin production from glycogen. Although H_2_ is an obligate co-product of the nitrogenase reaction, no H_2_ was produced in the initial simulations under dark N_2_-fixing conditions, contradicting experimental observations. Model examination revealed that all of the nitrogenase-generated H_2_ was utilized by hydrogenases to reduce NAD(P) and ferredoxin, which ultimately increased cyanophycin production. When the three hydrogenase reactions (HDH_1, HDH_2, and UPHYDR) were eliminated from the model, the predicted ratio of fixed N_2_ to consumed glycogen depended on the non-growth associated ATP requirement (NGAR), and was estimated to be 0.3 (NGAR = 2.8) or 0.67 (NGAR = 0) mole N_2_/mole glycogen, which was in accordance with an experimentally measured value of 0.51 [2]. Under this condition, the model predicted that H_2_ production would have same yields as fixed N_2_ (0.3 to 0.67 mole H_2_/mole glycogen) due to the stoichiometry of the nitrogenase reaction.

We also evaluated how fluxes through electron transfer reactions are affected by the nitrogenase flux under N_2_-fixing dark conditions. With glycogen being the sole source of reductant for both ATP-generating oxidative phosphorylation and N_2_ reduction, a balance between fluxes through respiratory pathways and nitrogenase reaction is needed. In the absence of the hydrogenase reactions, the model predicted that O_2_ reduction via COX, QOX, or Mehler reactions are required to consume NADH resulting from glycogen catabolism (Figure S2). The model predicts that the COX reaction is required to achieve the maximum N_2_ fixation rate since it generates more ATP than the QOX or Mehler pathways (∼9 O_2_ are needed per N_2_ fixed). This is consistent with the results from recent proteomic studies showing the CoxB1 (cce_1977) subunit of COX is more predominant during the dark [24], [25]. These results suggest terminal oxidases are important under dark N_2_-fixing conditions not only to generate an intracellular anaerobic environment for nitrogenase, but also to provide ATP for nitrogenase activity.

**Figure 2:**
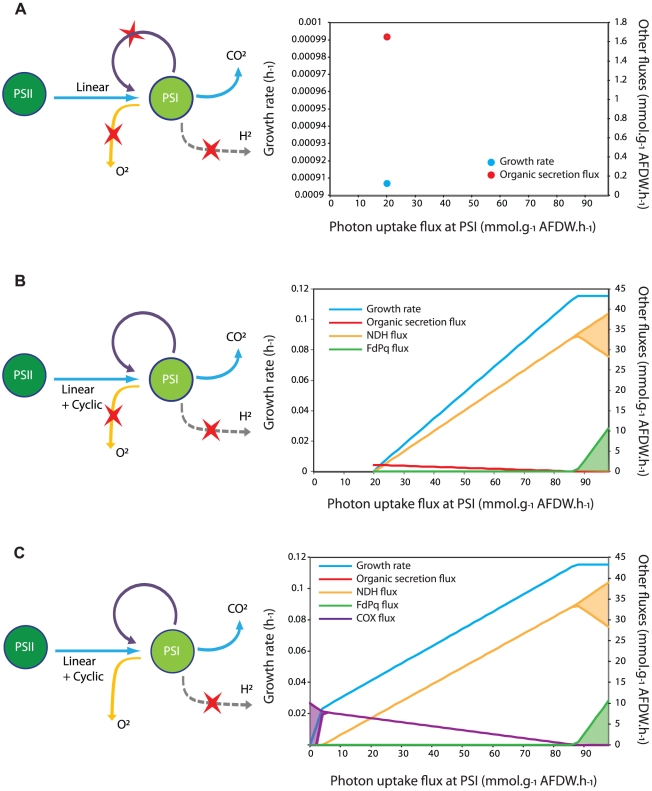
Impact of electron transport pathways on growth and metabolism of *Cyanothece* 51142. (A) Effects of removing cyclic photosynthesis (*via* NDH-1, NDH-2, FdPq, G3PD_PQ, and SUCD_PQ) and alternative reductant sinks (H_2_ production, COX, QOX, and Mehler reactions). (B) Effect of removing alternative reductant sinks but including all routes for cyclic photosynthesis. Shaded regions indicate that multiple flux values can achieve maximal growth rate. (C) All photosynthetic and respiratory electron flow routes operate, except H_2_ production.

As photosynthesis and respiratory electron transport chains are interconnected in cyanobacteria [26], these pathways were allowed to interact in the *i*Cce806 model. To perform model robustness analysis, we computationally explored the impact of key photosynthetic and respiratory pathways on growth rate and intracellular flux distributions under varying photon uptake flux for PS I, while the photon uptake flux for PS II was fixed at 20 mmol·g^−1^ AFDW·h^−1^ (Figure 2). First, the model was evaluated assuming only linear photosynthetic electron transfer. In this case, all alternative reductant sinks including the proton and O_2_ reduction as well as cyclic photosynthetic reactions around PS I were eliminated from the model (Figure 2A). Under this condition, growth only occurred at one value of photon uptake flux for PS I and extracellular organic products (ethanol, lactate and/or alanine with trace amounts of formate) would have to be secreted in order to generate enough ATP to support biomass production. Second, when cyclic photosynthetic reactions were added back, the photon uptake flux for PS I could vary with a fixed photon uptake flux for PS II, but significant amounts of extracellular products were still formed until the photon uptake flux for PS I exceeded ∼85 mmol·g^−1^ AFDW·h^−1^ (Figure 2B). No growth occurred unless PS I photon uptake flux was greater than or equal to the photon uptake flux for PS II. Only when the model was allowed to use both cyclic photosynthesis and O_2_ reduction reactions were no extracellular products predicted and the photon uptake flux for PS I could be less than that for PS II (Figure 2C). Since experimental data does not indicate that any by-products including H_2_ or organic acids are produced by *Cyanothece* 51142 at a detectable level during photoautotrophic growth with excess ammonium, a plausible mechanism for balancing growth through the generation of additional ATP may involve activity of the cytochrome oxidases.

### Effect of light quality on cellular growth and pathway utilization

**Figure 3:**
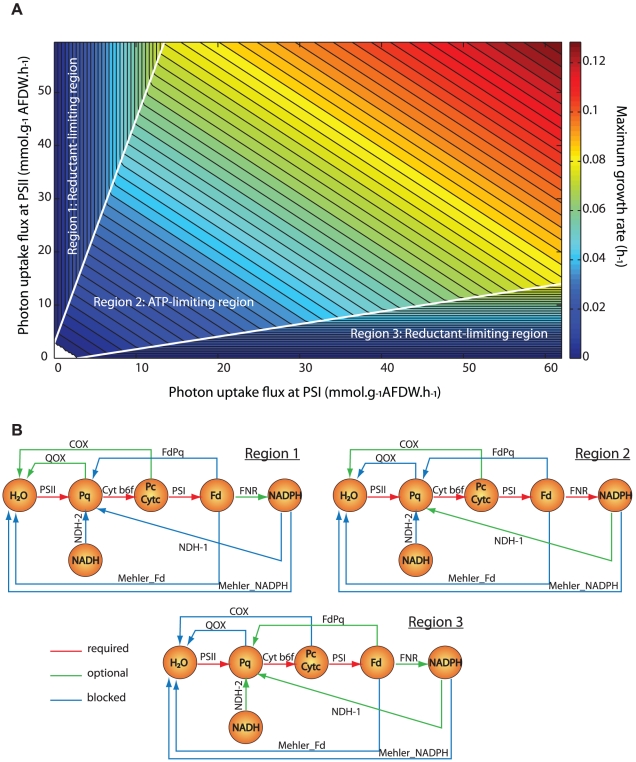
Predicted effects of varying photon uptake rates on growth and electron transport pathways. (A) 2-D phenotypic phase plane (PhPP) displaying maximum growth rates for varying photon uptake rates. The PhPP has 3 distinct regions – in regions 1 and 3, flux through a single photosystem limit growth rates, whereas in region 2 flux increases through either photosystem will increase growth rate. (B) Pathway maps of electron transfer reactions in different PhPP regions. PhPP flux variability analysis was performed to see which flux is always required (red arrows), optional (green arrows), and blocked (blue arrows) across each of the three PhPP regions.

The discrete representation of PS II- and PS I-mediated reactions and their interactions with multiple respiratory reactions in *i*Cce806 enabled further *in silico* analysis of growth and electron flux distributions under photoautotrophic conditions of variable light quality and intensity. In this case, the complete model was used to explore which reactions would be used to support maximal photoautotrophic growth rates for different levels of PS II and PS I photon uptake fluxes. To predict the corresponding growth rates under light-limited conditions, we constrained the photon uptake fluxes (ranging from 0 to 60 mmol·g^−1^ AFDW·h^−1^) through each photosystem. The resulting phenotypic phase plane (PhPP) contained three distinct regions (Figure 3A): in two regions growth was limited only by fluxes through PS II (region 1) or PS I (region 3), while in region 2 growth was limited by both PS II and PS I photon uptake fluxes (*i.e.*, increases in either flux would improve growth rate). By adding artificial ATP or NADPH generating reactions (ADP+HPO_4_+H→ATP+H_2_O and NADP+H→NADPH) to the model and analyzing changes in predicted maximal growth rates, we were able to identify that in regions 1 and 3 growth was NADPH/reductant-limited, while in region 2 it was limited by energy supply (Figure 3A).

To analyze the effect of photon uptake rates on electron flux distributions, we calculated the flux ranges using flux variance analysis (FVA) for all photosynthetic and respiration reactions within each PhPP region (Figure 3B). In this instance, PhPP FVA was run with constraints that restrict the model to a given region and to the maximum growth for each point in the region (in contrast, standard FVA is used at a single point in a region). Using PhPP FVA, we identified active (both minimum and maximum flux values are positive or negative), inactive/blocked (minimum and maximum fluxes are both zero), and optional (which could have at least one zero and one non-zero flux value somewhere in the region) reactions leading to optimal solutions in each PhPP region. This new analysis technique allowed classification of reaction usage across entire regions of the PhPP and is not restricted to fixed points within a region. While linear photosynthesis was active and Mehler reactions were blocked across the entire PhPP, there were differences in the usage of photosynthetic and respiratory reactions observed within all three regions (Figure 3B). Surprisingly, while generation of NADPH from reduced ferredoxin *via* linear photosynthesis is the key source of reductant, ferredoxin-NADP^+^ oxidoreductase (FNR) was predicted to be active in region 2, but optional in regions 1 and 3. Closer examination of *in silico* calculated electron flux distributions revealed that, in addition to FNR, the model utilized a cycle involving glutamine synthetase, glutamate synthase and transhydrogenase, resulting in ATP-driven NADPH production. In regions 1 and 3, the model predicts there is excess ATP, and so this cycle can be used instead of FNR to move electrons from ferredoxin to NADPH. However, this cycle is unlikely to be of any physiological relevance since there has been no experimental data supporting this route for making NADPH, and FNR is essential for photoautotrophic growth in unicellular cyanobacteria such as *Synechococcu*s 7002 [27]. Differences in the predicted usage of respiratory reactions were also found. In region 1, where growth is limited by the flux through PS I, at least one of the COX and QOX reactions must be active to oxidize excess electron carriers (Pc, Cyt *c_6_*, or Pq) generated from PS II. Similarly, in region 3 under PS II flux limitation, excess electron carriers (Pq, Fd) must be reduced *via* NDH-1 or –2 or ferredoxin-dependent cyclic electron transfer (FdPq). Conversely, due to ATP limitation in region 2, the model favored reactions with higher proton pumping capacities and so both the QOX and FdPq reactions were inactive. The usage of COX was optional in region 2 and depended on photon uptake rates (*e.g.*, COX reaction was inactive at the boundary between regions 2 and 3).

The model predictions (Figure 3A) were compared to batch growth experiments in the LED-photobioreactor which allowed instantaneous measurements of initial growth and photon uptake rates by *Cyanothece* 51142 cells exposed to different intensities and ratios of 630 and 680 nm light (Table 1). When *Cyanothece* 51142 cultures were illuminated with both 630 nm and 680 nm light, initial growth rates generally correlated with the total photon flux through PS II and PS I, with higher growth rates observed at 80 mmol·g^−1^ AFDW·h^−1^ total photon flux and 630 nm∶680 nm light ratio of 2∶1. When cultures were exposed to only a single wavelength of light (batch experiments 6–10), *i.e.*, either 630 or 680 nm, *Cyanothece* 51142 cells displayed a similar trend with higher growth rates observed at higher photon flux intensities. The predicted growth rates were within 7% of the experimentally measured values, except for the two cases where single 630 nm wavelength irradiances were used (Table 1). The reasons for this are unclear but may be due to other physiological and/or biochemical phenomena such as state transitions that are not contained within the model but are operating *in vivo*.

**Table 1:** Comparison of growth rates predicted by simulation model to those experimentally measured in batch cultures.

Experiment	Photon uptake rate* at 630 nm, mmol•g^−1^ AFDW•h^−1^	Photon uptake rate* at 680 nm, mmol•g^−1^ AFDW•h^−1^	Total photon uptake rate mmol•g^−1^ AFDW•h^−1^	Experimentally measured growth rate, h^−1^	Predicted growth rate, h^−1^
1#	19.0±1.1	15.5±0.9	34.5	0.035±0.0068	0.035±0.0022
2#	15.6±1.1	26.0±1.6	41.6	0.041±0.0076	0.043±0.0032
3#	33.4±1.0	13.6±0.4	47.0	0.051±0.0053	0.049±0.0018
4#	34.6±1.4	35.0±1.3	69.6	0.079±0.0062	0.074±0.0033
5#	53.6±2.8	26.4±1.0	80.0	0.080±0.0052	0.085±0.0044
6**	0	32.1±2.0	32.1	0.032±0.0012	0.032±0.0025
7**	0	33.0±2.1	33.0	0.037±0.00014	0.033±0.0026
8**	0	37.2±1.5	37.2	0.040±0.00032	0.038±0.0017
9**	21.1±1.7	0	21.1	0.016±0.010	0.020±0.0021
10**	28.0±1.7	0	28.0	0.036±0.014	0.028±0.0021

*: Average and standard deviation of the instantaneously measured growth rate and photon uptake rates were calculated over the first 5 hours. (See Text S1 for more detail about batch growth rate simulation).

**: For computational predictions of the growth rate for batches 6–10, the total photon uptake flux measurements at 630 nm and 680 nm was used to constrain the total photon uptake flux in the model (EX_photon PSI_e+EX_photon PSII_e).

# Experimental photon uptake and growth rates from batches 1–5 were used to calculate ATP requirement parameters GAR and NGAR.

Data from these batch experiments (batch experiments 1–5, Table 1) were also used to estimate the growth (GAR) and non-growth (NGAR) associated ATP requirements. NGAR is the amount of energy spent to maintain the cell (i.e., maintenance energy). GAR is defined as energy expenditures used on protein and mRNA turnover or repair, proton leakage, and maintenance of membrane integrity; it does not include ATP spent on polymerization reactions, which are already accounted for in the macromolecular synthesis pathways of the network. The time-averaged growth and photon uptake rates were used to constrain the model and the maximal amount of ATP hydrolysis was calculated (Figure S3) for each batch experiment. A plot of growth rate versus maximum ATP hydrolysis flux was generated and a linear fit used to estimate the GAR and NGAR values [28]. Specifically, the slope of the fitted line is the GAR (544 mmol·g^−1^ AFDW·h^−1^), and the y-intercept is NGAR (2.8 mmol·g^−1^ AFDW·h^−1^). The estimated GAR value is significantly higher than those reported from other bacteria [29]; however, these model estimates assume that all absorbed photons lead to photosynthetic fluxes (100% quantum efficiency) and that the overall efficiency of ATP production via all electron transfer reactions (photosynthetic and respiratory) are accurate. Depending on the growth condition the quantum yields can change, and for *Cyanothece* 51142 this value was reported to be between ∼70–100% for photoautotrophic growth [23]. Upon further analysis, we found the estimated *Cyanothece* ATP requirements were most sensitive to reductions in quantum efficiency and the amount of ATP generated by photosynthesis and respiration (Table S4). Since neither quantum efficiency nor combined photosynthetic and respiratory ATP production were experimentally measured for *Cyanothece* 51142, the original estimates, GAR = 544 and NGAR = 2.8 were used in all growth simulations.

### Experimental analysis of ammonium- and light-limited growth

**Figure 4:**
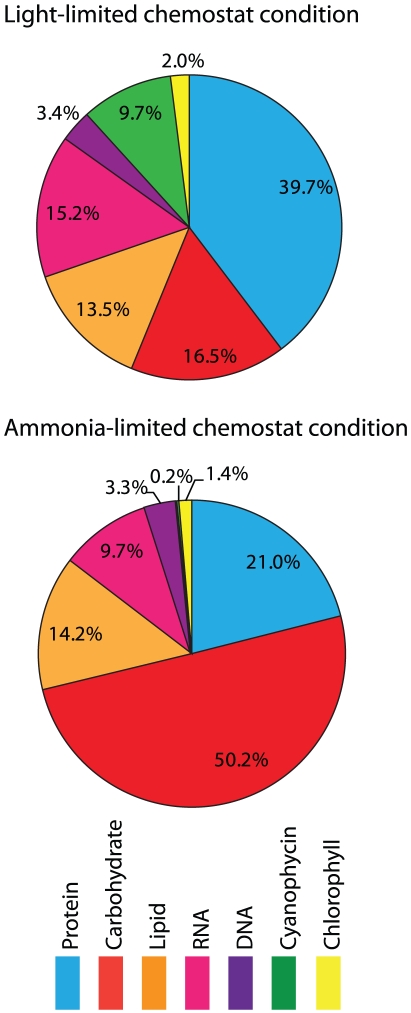
Effect of nutrient limitation on biomass composition (normalized to ash-free dry weight).

Chemostat cultures grown under light and ammonium limitations were used to calculate metabolic fluxes and further understand reductant partitioning pathways in *Cyanothece* 51142. The differences in biomass composition between these growth conditions indicated a major shift in carbon partitioning pathways (Figure 4; and Table S5). In ammonium limited cultures, carbohydrates comprised almost half of cell biomass; in contrast, under light limitation, *Cyanothece* 51142 cells contained higher amounts of protein, nucleic acids, and approximately 10% cyanophycin. The quantitative biomass composition measurements were used to generate two separate biomass equations for the metabolic model; experimentally measured growth rate, photon uptake rates, and O_2_ production rates were included as additional model constraints (Table S6, in this case no mRNA or protein expression data is used by the model). Using FBA and through minimization of the overall flux magnitude, we calculated representative flux distributions under light and ammonium limitations (values listed in Table S1). As expected, changes in flux values were consistent with differences in measured biomass compositions used in the simulations: under light limitation, fluxes increased for reactions involved in biosynthesis of amino acids, nucleotides and cyanophycin, while ammonium limitation resulted in flux increases for glycogen biosynthesis.

Comparisons of global transcriptome profiles displayed by *Cyanothece* 51142 during ammonium- and light-limited chemostat growth also reflected the rewiring of cellular metabolism (Table S7). Under ammonium limitation, significant increase in relative mRNA abundances was observed for genes involved in N_2_ fixation (cce_0198, cce_0545–0579), iron acquisition (cce_0032–0033, cce_1951, cce_2632–2635), respiratory electron transport (cce_1665, cce_3410–3411, cce_4108–4111, cce_4814–4815) as well as peptide transport, synthesis, and protein repair (cce_0392, cce_1720, cce_3033, cce_3054–3055, cce_3073–3075). Among the most highly expressed genes in ammonium-limited *Cyanothece* 51142 cells was the one encoding 6-phosphogluconate dehydrogenase (cce_3746), a key PPP enzyme. Under light limitation, the major changes in the transcriptome of *Cyanothece* 51142 included upregulation of genes encoding: components of the photosynthetic apparatus and electron transport chain (cce_0776, cce_0989–0990, cce_1289, cce_2485, cce_2959, cce_3176, cce_3963); pigment biosynthesis (cce_0920, cce_1954, cce_2652–2656, cce_2908, cce_4532–4534); CO_2_ uptake and fixation machinery (cce_0605, cce_3164–3166, cce_4279–4281); ATP synthase (cce_2812, cce_ 4485–4489), and protein synthesis machinery (cce_ 4016–4030) (Table S7).

Global proteome profiles of *Cyanothece* 51142 corroborated the shifts in gene expression (Table S8). The abundance of proteins from central metabolism (glycolysis, TCA, and pentose phosphate pathway) all had significant differences between cells grown under ammonium- and light-limited conditions. Enzymes of the oxidative PPP branch, namely glucose-6-phosphate dehydrogenase (cce_2535–2536), 6-phosphogluconolactonase (cce_4743) and 6-phosphogluconate dehydrogenase (cce_3746), showed increased abundances under ammonium limited conditions. Similarly, two-fold increase in abundance levels was observed for gluconeogenesis proteins, including fructose 1,6-bisphosphatase (cce_4758), glucose-6-phosphate isomerase (cce_0666), glyceraldehyde-3-phosphate dehydrogenase (cce_3612), and phosphoglycerate kinase (cce_4219). In contrast, relative abundances of proteins catalyzing the conversion of glycerate-3P to pyruvate (cce_1789 and cce_2454) were unchanged or up-regulated (pyruvate kinase cce_3420) in light-limited cells. Consistent with the results from global mRNA profiles was the up-regulation of *Cyanothece* 51142 proteins involved in photosynthesis and carbon fixation under light-limited conditions (Table S8). Notably, two key components involved in the electron transfer to PS I, namely plastocyanin (cce_0590) and cytochrome *b*_6_ (cce_1383), displayed elevated peptide abundances in light-limited cells.

### Using experimental measurements and *in silico* mutagenesis to restrict the range of predicted flux distributions

**Figure 5:**
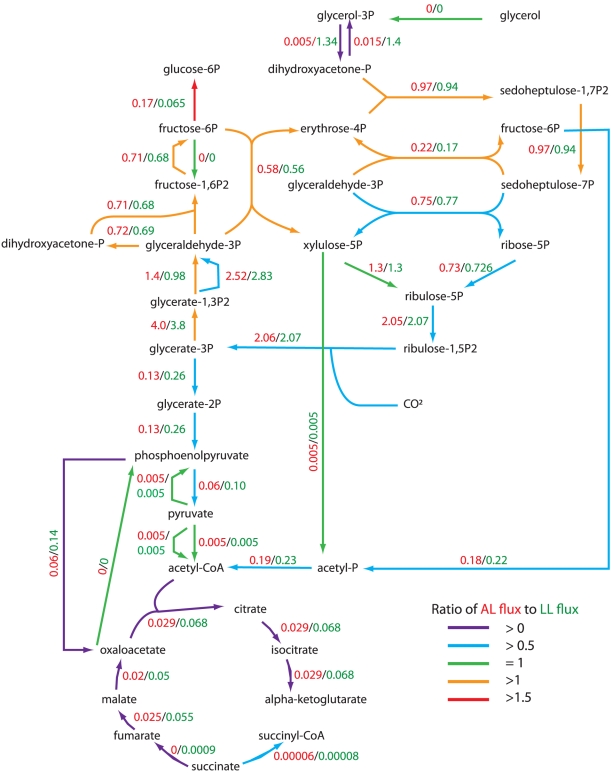
Predicted chemostat flux distributions in central metabolism including transcriptome and proteome data as constraints. The flux values (mmol·g^−1^ AFDW·h^−1^) are those where the flux distribution best matches the transcriptome and proteome data (TPD) while also minimizing the magnitude of all fluxes in the network. The flux values in red and green represent ammonia-limited (AL) and light-limited (LL) conditions, respectively. Arrow colors indicate relative flux ratios between AL and LL conditions.

Since there may be more than one flux distribution that is consistent with the experimentally measured rates of growth, photon uptake, and O_2_ production we used FVA to identify required (flux must be non-zero), optional (flux may or may not be zero), or inactive (flux must be zero) reactions under light- and ammonium-limited growth conditions. As our initial simulations (Table 2) produced a large number of optional reactions (170 out of 667 for both growth conditions), that represent uncertainty regarding usage, we subsequently used the transcriptome and proteome data (TPD) to further constrain the model. Using a modification to a previously developed approach [30], we obtained a flux distribution that was consistent with measured rates and TPD while reducing the overall flux magnitude (Table S1). In this analysis, flux was favored through reactions for which proteins were detected and disfavored through reactions associated with undetected proteins and transcriptome data less than a given threshold (e.g., log_2_ of mRNA expression level is less than 8). The model constrained by TPD predicted that the majority of reactions in central metabolism would be active under both chemostat conditions (Figure 5). In addition, we subsequently applied FVA employing additional constraints arising from the TPD. Comparison between FVA results with and without TPD constraints demonstrated a significant decrease in the number of ambiguities (the optional reaction set) when TPD is used (Table 2).

**Table 2:** Flux variability analysis for model simulations in light-limited and ammonium-limited chemostat conditions.

	With protein and mRNA expression data		Without protein and mRNA expression data
Category	Light-limited	NH_4_- limited	Both conditions
Required reactions	364	366	287
Optional reactions	74	76	170
Inactive reactions	229	225	210

**Figure 6:**
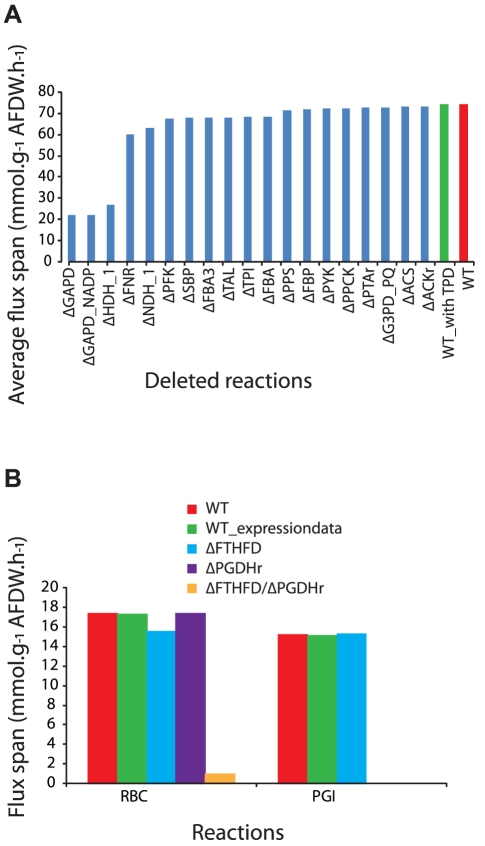
Effects of *in silico* reaction deletions on flux spans under light-limited conditions. (A) Effects of deletions are compared to the cases where no reactions were deleted (red bar), or TPD were used as constraints (green bar). The values represent the average flux span across all reactions in central metabolism. Only deletions which lower the flux span by at least >1 mmol·g^−1^ AFDW·h^−1^ are presented. (B) Changes in flux spans for specific reactions catalyzed by ribulose bisphosphate carboxylase (RBC) and phosphoglucose isomerase (PGI) between simulations that (i) use TPD data as a constraint (green bars), (ii) delete single reactions (blue and purple bars), (iii) delete two reactions (yellow bar) or (iv) impose no additional constraints (red bars). Reaction abbreviations match those listed in Table S1.

While the number of optional reactions was reduced by incorporating TPD into the model, the flux spans (difference between maximum and minimum values) of individual fluxes was still large (>30 mmol·g^−1^ AFDW·h^−1^ for some central metabolic reactions, Table S1). These large flux spans could arise from cycles or alternative pathways in the model, and deleting these features from the model could subsequently reduce the flux spans. FVA was repeated using measured growth, photon uptake, and O_2_ release rates under light-limited conditions as constraints and with optional reactions were deleted (similar results were found for ammonia limited conditions, data not shown). Flux spans for reactions in central metabolism (Figure 5) were then calculated for a series of single or double reaction deletions *in silico*. The purpose of this analysis was to identify those reactions that exert the greatest impact on the flux span in central metabolism (Figure 6A). Single deletions of glyceraldehyde-3-phosphate dehydrogenase (GAPD or GAPD_NADP) or hydrogenase (HDH_1) reduced the average central metabolic flux span the most (from 74 to 22 mmol·g^−1^ AFDW·h^−1^). Other single deletions with significant effects included FNR and NDH-1, which are involved in photosynthesis and respiration. The reaction deletions shown in Figure 6A all had a larger impact on reducing average central metabolic flux span than did imposition of constraints based on TPD. There were cases where single deletions had large effects on other specific reactions, but only modest effects on overall central metabolic flux spans. For example, a single deletion in phosphogluconate dehydrogenase (PGDHr) reduced the span for glucose-6-phosphate isomerase flux (PGI) to 0 (Figure 6B), but only reduced the average central metabolic flux span by ∼0.7 mmol·g^−1^ AFDW·h^−1^. The *in silico* analysis of double reaction deletions did not yield any new double deletions that would reduce the average central metabolic flux span significantly. However, some double deletions strategies did reduce flux spans of individual reactions.

## Discussion

Several cyanobacterial metabolic models (all for *Synechocystis* PCC 6803) have been published, which represented photosynthesis as two lumped reactions [12], [31] for linear (PSII, Cyt *b_6_f*, PSI, and FNR) and cyclic (PS I and Cyt *b_6_f*) pathways. In this study, we modeled photosynthesis as a larger set of separate reactions [13] as this structuring allowed analysis of the effects of different illumination on the production and partitioning of reductant through photosynthetic and respiratory reactions, as well as the contribution of different electron transfer pathways to growth. Our PhPP FVA results showed how different photosynthetic and respiratory electron transport chain components are used to maximize biomass production under different lighting regimes. It was not surprising that linear photosynthesis was active in all three regions because the cell needs photons from both PSI and PSII to grow under photoautotrophic conditions. However, the Mehler reactions were inactive in all three regions when we only consider maximal growth rate solutions. In regions 1 and 3, reducing equivalents (e.g., NADPH) limit growth and the Mehler reactions would lower the amount of reducing equivalents available for growth. The Mehler reactions are less energetically efficient than NADH dehydrogenase and cytochrome oxidase so the model would not use them in region 2, where ATP is limiting. So while the Mehler reactions can carry flux in the model, using these reactions lowers the maximum growth rate making them inactive (blocked reactions) in our PhPP analysis. A recent study showed that the Mehler reactions are operational in *Synechocystis* sp. PCC 6803, serving as a sink for excess electrons [32]. These reactions are also likely to be active in *Cyanothece* 51142, since the associated proteins were detected in the proteomic data (Table S8). As a result the model only predicted non-zero Mehler fluxes when the proteomic data were used to constrain the model (Table S1).

In the absence of cyclic photosynthesis, other products including water (produced by COX, QOX or Mehler reactions), H_2_ (*via* hydrogenase), or small organic compounds (alanine, ethanol, lactate and formate) were predicted to be necessary in order to balance the electrons and ATP needed to support growth. In the presence of linear and cyclic photosynthesis reactions, these products must also be produced unless significant amounts of cyclic photosynthesis occurs (>3 times the amount of linear photosynthesis). Since H_2_ and small organic compounds are not generally produced under photoautotrophic conditions with excess ammonium, any additional energy is most likely supplied by cytochrome oxidase activities that reduce photosynthetically produced O_2_. Interestingly, in the absence of cytochrome oxidase activities in the model, the PS I fluxes must always be greater than or equal to the PS II fluxes. It was shown that the marine cyanobacteriium *Synechococcus* has a PS I/PS II protein ratio >1, which has been explained as a mechanism to protect PS II from photo-damage [33]. Under conditions with high levels of PS II activity, cytochrome oxidase activity may ensure an adequate supply of oxidized plastoquinone (needed for PS II) and reduce O_2_ concentrations to limit photorespiration.

Similarly, cyclic electron flow *via* NADH dehydrogenase- or ferredoxin-dependent routes have also been experimentally demonstrated to play important roles in balancing the amount of NADPH and ATP produced via photosynthesis. *Synechocystis* 6803 mutants lacking *ndhD* genes (encoding subunits of NDH-1) had significantly lower cyclic photosynthesis activity [34]. Although the mechanism of electron transfer from ferredoxin to the plastoquinone pool (without using NDH) is still unclear, its activity has been demonstrated in green algae [35] and higher plants [36]. Our computational simulations also showed that, under light-limited photoautotrophic conditions, cyclic electron transfer involving NADH dehydrogenase (NDH-1) is needed for maximal growth if ATP (rather than NADPH) is limiting. In an environment where PS I photon availability is high relative to PS II, cyclic electron transport is needed (Figure 2) to increase availability of PS I substrates (reduced PC or Cyt *c*_6_) and protect against photo-damage. Cyclic electron flow has been experimentally shown to help protect the photosynthetic apparatus from photo-damage [37]–[39]

In addition to studying the interactions between components of the photosynthetic and respiratory components computationally, we also experimentally evaluated cells grown under continuous light conditions in light- and ammonia-limited chemostats. The measured 630 nm and 680 nm photon uptake and O_2_ production rates suggests that reductant was being directed towards O_2_ *via* the Mehler, QOX, and/or COX reactions. In both chemostat conditions, the model predicted that steady-state growth rate could have been achieved using lower photon uptake rates by decreasing the amount of reductant generated by PS II that was predicted to reduce O_2_.

A limitation to flux balance analysis is that a wide range of flux values may be consistent with the constraints in the computational model. An iterative application of computational and experimental methods is an important strategy to improve the comprehensive understanding of cyanobacterial metabolism. We have begun to apply this iterative approach, by including mRNA and protein expression datasets as additional constraints beyond biomass composition and physiological rate measurements. Experimentally-measured TPD were successfully used to further constrain the model, and thereby reduce uncertainty and increase the number of required (that is, metabolically active) reactions (Table 2). However, there remained discrepancies in that the model did not predict flux through all reactions for which proteins were experimentally detected. Such discrepancies can be used to subsequently improve the model with previously developed approaches [40]–[42]. For example, an earlier version of the model did not predict flux through proline oxidase, even though proteome data demonstrated that proline oxidase was synthesized. This prediction arose because the model did not contain a reaction in which FADH_2_ (a product of the proline oxidase reaction) could be reoxidized to FAD. After experimental confirmation that proline can be used as a nitrogen source (implying activity of proline oxidase) by *Cyanothece* 51142 (data not shown), a FADH_2_ recycling reaction was included in the final *i*Cce806 model.

Even with these additional TPD constraints, a wide range of flux values remained feasible (Figure 6). We should note that we did not take real enzymatic activities into account (which can be affected by post-translational modifications), as we did not have this type of data for the two conditions examined. Such data, if available, could be used as additional factors for determining whether to favor or disfavor fluxes through associated reactions (See Material and Methods). Other constraint-based methods for incorporating gene expression data use similar Boolean on/off type of constraints to restrict fluxes [30], [43], [44] and would be expected to yield results similar to those described herein. Thus, novel computational methods which can more quantitatively constrain the metabolic flux values are still needed. The strategy of evaluating fluxes for reaction deletions *in silico* can be used to identify knockout mutants that can potentially improve the resolution of intracellular flux distributions. A flux that is well resolved would have a small span meaning we can more definitively state its value. If the mutants show no growth defects then the corresponding reactions may not be used under the conditions tested, or alternative pathways not included in the model may occur. Either way, this information could be used to better resolve the intracellular flux distribution or improve the metabolic model. For *Cyanothece* 51142, this would require development of a genetic system (such a system already exists for another *Cyanothece* strain [45]) as experiments with mutants would have the most potential to improve resolution of central metabolic fluxes during photoautotrophic growth. Also, as a complement to the *in silico* reaction knockouts that our simulations predict would reduce the flux spans associated with central metabolic reactions, the photobioreactor employed here provides a system whereby cultivation conditions can be rigorously controlled and some aspects of physiological state monitored continuously. In addition, cells from steady-state or perturbed cultures can be interrogated via physiological or biochemical analyses to experimentally test the predictions of the computational models for wild type or mutants. As the number of available cyanobacterial models continues to grow, cross-species physiological, genomic, and metabolic comparisons will enable the identification of core networks and contribute towards improving our understanding of metabolic processes in cyanobacteria.

## Material and Methods

### Culture and growth conditions

*Cyanothece* 51142 was grown in modified ASP2 medium [46] amended with 0.75 mM K_2_HPO_4_, 0.03 mM FeCl_3_•6H_2_O, and 17 mM NH_4_Cl which substituted NaNO_3_ as the nitrogen source. Routinely, the cells were maintained under continuous white light illumination (50 µmol photons·m^−2^·s^−1^) in 1-L Roux bottles sparged with CO_2_-enriched air (0.3% vol/vol). Culture purity was confirmed by plating on DIFCO Bacto Tryptic Soy Broth and DIFCO Luria-Bertani solid media (BD Diagnostic Systems, Franklin Lakes, NJ) as well as by phase contrast or acridine orange fluorescent microscopy.

### Controlled cultivation

Controlled batch and chemostat cultures of *Cyanothece* 51142 were grown in a 7.5-L borosilicate glass vessel operated at 5.5-L working volume under the control of New Brunswick Bioflo 3000 bench-top bioreactor (New Brunswick Scientific, Edison, NJ). The vessel was housed in a custom-made black anodized aluminum enclosure equipped with light-emitting diodes (LED) generating 680 nm and 630 nm light for the preferential excitation of chlorophyll *a* and phycobilin pigments, respectively. Built-in sensors allowed for automatic adjustment of incident and transmitted light intensities using custom-designed control module. Both hardware and software components of the LED enclosure and the control module were developed at Pacific Northwest National Laboratory (US Patent Application # 20100062483; http://appft1.uspto.gov). All experiments were carried out under continuous illumination in modified ASP2 medium sparged with CO_2_-enriched argon (Ar) (0.2% vol/vol). Agitation, temperature, pH, and gas flow rates were maintained at 250 rpm, 30°C, 7.4, and 2.8 L/min, respectively. Incoming and off-gas composition was constantly monitored by an in-line mass spectrometry based gas analyzer MGA iSCAN (Hamilton Sundstrand, Pomona CA). Cell density was monitored spectrophotometrically at 625, 678, and 730 nm.

To establish a light-limited chemostat culture, the photobioreactor was inoculated with 10 mL of mid-log phase *Cyanothece* 51142 cells and maintained as a batch culture under 630 nm and 680 nm illumination at 40 and 70 µmol photon·m^−2^·s^−1^, respectively, until the culture reached late logarithmic stage. Chemostat mode was initiated by continuous inflow of medium at a dilution rate of 0.05 hr^−1^ that resulted in a steady-state optical density (OD_730_) of 0.20. Similarly, a nitrogen-limited continuous culture of *Cyanothece* 51142 was established using low-nitrogen ASP medium containing 0.75 mM NH_4_^+^. The ammonium-limited chemostat was maintained under identical operating conditions in regard to the culture dilution rate and optical density under incident light at 38.5 and 73.5 µmol photon·m^−2^·s^−1^ for 630 nm and 680 nm wave lengths, respectively).

The light uptake fluxes (mmol·g^−1^ AFDW·h^−1^) were determined by multiplying the light consumption rates (µmol photon m^−2^s^−1^) by the surface area of cell culture exposed to light (m^2^) and dividing by the amount of biomass in the reactor (g AFDW). The light consumption rates were determined by subtracting the transmitted light intensity from the values of incident light intensity after corrections were made for the abiotic consumption of light to account for the gas bubbles and probes in the reactor. Cells in the 5.5 L working volume were assumed to be equally exposed to the light at all times. Based on the inner diameter and height of the liquid culture at working volume, the surface area was 0.1403 m^2^. The amount of biomass in the reactor was determined from the working volume and biomass concentrations.

### Analytical methods

Biomass ash-free dry weight (AFDW) was measured using centrifuged (11,000× g, 4°C) cell pellets as described previously [29]. Total protein, reducing carbohydrates, RNA, and DNA were assayed using standard analytical techniques [47]–[49]. The total lipid fraction was measured gravimetrically after an extraction from a known volume of freeze-dried culture using previously published methodology [50]. Total reducing carbohydrates were quantified using the anthrone method [51] with glycogen as the standard. Chlorophyll concentrations were measured as described elsewhere [52], [53]. Amino acid composition was analyzed in acid-phenol hydrolyzed samples prepared using Eldex hydrolysis/derivatization station (Eldex Laboratories, Inc., Napa, CA) [29]. The derivatized samples were resolved on a 4-µm AccQ-Tag Nova-Pak C-18 column (3.9 mm×150 mm, Waters Corp., Milford, MA, USA), eluted using a linear gradient of acetonitrile (from 1.2% to 4.2% over 15 min.; from 4.2% to 6% over 4 min.; from 6% to 20% over 12 min.; at 20% over 1 min.; from 20% to 60% over 1 min.) with a flow rate of 1.0 ml/min at 37°C, and detected at 254 nm (HPLC system and UV detector by Shimadzu, Tokyo, Japan). Cyanophycin was estimated based on relative amino acid values and total protein measurements (see Text S1 for details).

### Microarray expression analysis

Previously developed whole-genome oligonucleotide microarrays of *Cyanothece* 51142 [7] were manufactured by Agilent Technologies (Santa Clara, CA). RNA isolation, labeling, hybridization, and data analysis were performed by MOgene, LC (St. Louis, MO) using published protocols [7].

### Proteomic analysis

Cell lysis and tryptic digestion followed a previously described “global protein preparation” scheme [54]. A reference peptide database was prepared using strong cation exchange fractionation (10 fractions) of a portion of each global digest, as previously reported [55]–[57]. The methods for capillary liquid chromatography and mass spectrometry have been described in detail elsewhere [54], [58], [59]. Here, the HPLC mobile phase was 0.1% formic acid in water (A) and 0.1% formic acid in acetonitrile (B). A Finnigan LTQ ion trap mass spectrometer (ThermoFinnigan, San Jose, CA) was used for MS/MS analysis of SCX fractions and an LTQ-Orbitrap (Thermo) was used for high-resolution MS analysis of the global unfractionated samples. Each of the 10 SCX fractions was analyzed once, while each global digest was injected four times.

To build an accurate mass and time (AMT) tag database, SEQUEST analysis software was used to match the MS/MS fragmentation spectra to sequences from the annotation of the *Cyanothece* 51142 proteome [6]. Peptide identifications from the SCX fractions were combined with identifications from unfractionated samples to create a reference database of calculated mass and normalized elution time for each identified peptide. This database was used for subsequent high-sensitivity, high-throughput analysis of *Cyanothece* 51142 samples using the AMT tag approach [60]. LC-MS features from the unfractionated global samples were matched to the rich database built from the fractionated samples to give accurate peptide IDs. The area of each LC elution peak was used as a measure of peptide abundance.

Data from the AMT output were imported into the software MDART (Burnum et al., unpublished results), for filtering using a mass error tolerance of <5 parts per million, delta match score >0 (a measure of peptide uniqueness), match score >−1, and absolute normalized elution time error <10,000. The resulting 7450 peptides were imported into the software tool DAnTE [61] for further filtering and analysis. Peptide abundances were transformed to log base 2 and mean-centered. A linear regression-based normalization method available in DAnTE was then applied within each replicate category. Peptide abundances were used to infer the corresponding protein abundances through the ‘Rrollup’ algorithm in DAnTE [61]. During the Rrollup step, peptides were excluded if not present in at least 3 of the eight datasets, and Grubbs' outlier test was applied with a *P*-value cutoff of 0.05 to further remove outlying peptides. For increased confidence in protein identifications, each protein was required to be identified by at least 2 unique peptides, resulting in a total of 865 proteins. The minimum observed relative protein abundance value (14,465) was imputed as a crude surrogate for missing data for statistical calculations. Statistical differences between the two samples (4 technical replicates of each) were determined using ANOVA with a *P*-value cutoff of 0.05 (q<0.03) in DAnTE [61].

### Metabolic network reconstruction: *iCce806*

A draft metabolic network of *Cyanothece* 51142 was reconstructed in SimPheny (Genomatica, San Diego, CA) using a previously described automated model-building process [62]. Metabolic reactions and gene-to-protein-to-reaction (GPR) associations from other models were incorporated into the reconstruction if good BLAST hits could be found between genes in *Cyanothece* 51142 and genes in other modeled organisms. Additional reactions were added as necessary to produce known biomass constituents or utilize known nutrients; detailed literature, database, and BLAST searches were then carried out to find genes encoding these reactions in *Cyanothece* 51142 genome. This resulted in several new GPR associations that were incorporated into the reconstruction.

### Constraint-based analysis of flux distributions

Based on the metabolic reconstruction, a constraint-based metabolic model for *Cyanothece* 51142 was developed as described in [63]. Fluxes are limited based on several different types of constraints: steady-state mass balance constraints (Eq. 1), enzyme capacity and thermodynamic constraints (Eq. 2) [10], given by:
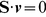
(1)

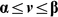
(2)where **S** is a stoichiometric matrix for the reaction network, ***v*** is a flux vector, and **α** and **β** are parameters that limit the capacity and directionality of individual reactions. Flux balance analysis (FBA) uses these constraints to identify a flux distribution which maximizes or minimizes an objective function, such as growth rate [10]. Flux variability analysis (FVA) can also be used to determine the range of values each flux can take that are consistent with Eq. 1 and 2, by maximizing and minimizing each flux individually [64].

To further constrain the models based on mRNA or protein expression data, a modified version of the method developed by Shlomi *et al.* [30] was used. Here, we identified a single flux distribution that best agreed with measured transcriptome and proteome data (TPD) and minimized flux usage. Reactions with experimentally measured fluxes belong to set R_E_ (which included biomass production and exchange fluxes for oxygen, 630 nm and 680 nm photons) and were constrained to their measured values. Reactions associated with detected proteins were included in the high reaction set (R_H_). Reactions associated with undetected proteins and genes with low mRNA expression levels (whose mRNA expression was less than the lowest mRNA expression of detected proteins) were included in the low reaction set (R_L_). The method finds a flux distribution that maximizes the number of active reactions (*_v_*_≠0_) and inactive reactions (*_v_*_ = 0_) in reaction sets R_H_ and R_L_, respectively. For reactions in set R_H_, binary variables *x* and *y* indicate whether a reaction is active, meaning its flux is greater than a positive threshold ε (*x* = 0 and *y* = 1), or smaller than a negative threshold -ε (*x* = 1 and *y* = 0) for reversible reactions. If both *x* and *y* are zero then the reaction is inactive and its flux value is zero. Likewise, a binary variable *z* is used for reactions in set R_L_ such that if *z* = 1 then the reaction is inactive (*v* = 0). The original method [30] has alternate solutions, which can contain unrealistically high flux values due to the presence of cycles (e.g., futile cycles and circulations) in the network. To identify a solution that minimizes the use of these cycles, the objective function was modified to also minimize the sum of squared fluxes through the network.

The mixed integer quadratic programming formulation to identify a flux distribution that best matches TPD while minimizing flux magnitude is given below (Eq. 3).
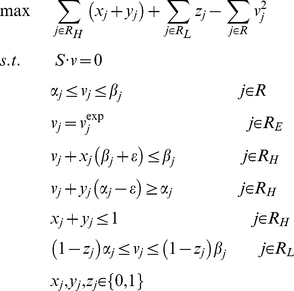
(3)Additionally, to find the flux ranges consistent with the TPD, flux variability analysis (FVA) was performed by minimizing and maximizing the flux through each reaction in the network. In these FVA simulations, the same constraints described above were included and the binary variables (*x*, *y*, and *z*) were further constrained by their optimal values (*x* ^opt^, *y* ^opt^, and *z* ^opt^) found in the original problem (formulation below, Eq. 4). In this study, all model simulations were performed in GAMS software (General Algebraic Modeling System, GAMS Development Corporation, Washington, D.C.
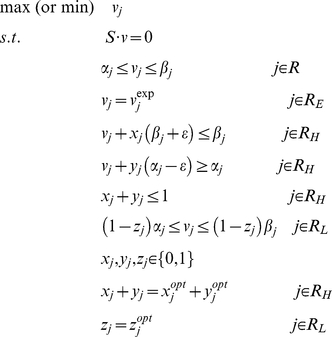
(4)


## Supporting Information

Dataset S1 **SBML file of the model** ***i*** **Cce806 which stores reactions and GPR associations.**
(XML)

Figure S1 ***In silico*** **predictions for biomass yields under photoautotrophic, heterotrophic and photoheterotrophic conditions.** Comparison of maximal biomass yields per g of C substrate when different nitrogen sources are used. Under photoautotrophic conditions CO_2_ uptake flux was fixed at 1 mmol.g^−1^ AFDW.h^−1^ and photon uptake fluxes at PSI and PSII were fixed at 10 mmol•g^−1^ AFDW•h^−1^. In the heterotrophic simulations glycerol was the limiting nutrient. Glycerol uptake was fixed at 1 mmol•g^−1^ AFDW•h^−1^ and maximal biomass yields were calculated under dark conditions. In photoheterotrophic simulations both glycerol and light were limiting (so an increase in either would improve growth rates). In this case, glycerol uptake rate was fixed at 1 mmol•g^−1^ AFDW•h^−1^, while photon uptake fluxes for PSI and PSII were both fixed at 10 mmol•g^−1^ AFDW•h^−1^. Since light was limiting in the photoheterotrophic condition CO_2_ was predicted to be secreted and not used as an additional carbon source.
(EPS)

Figure S2 **Effects of distribution of fluxes through electron transport chains (ETC) on nitrogenase flux.** Nitrogen fixation (nitrogenase) flux was varied while fluxes through ETC reactions were maximized and minimized under dark N_2_-fixing condition with all hydrogenase reactions eliminated from the model. Under this condition the amount of H_2_ produced is equal to the nitrogenase flux. A glycogen demand reaction was added to the model (→Glycogen; allowing for glycogen consumption) and its flux was limited to 0.171 mmol•g^−1^ AFDW•h^−1^. A) Effects of distribution of fluxes through cytochrome c oxidases (COX) and cytochrome-quinol oxidases (QOX) on nitrogenase flux. B) Effects of distribution of total flux through COX and QOX, and flux through Mehler reactions on nitrogenase flux. C) Effects of distribution of fluxes through NADH dehydrogenase reactions (NDH) and Fd-dependent cyclic reaction (FdPq) on nitrogenase flux. Shaded regions indicate ETC reactions can have multiple values for a particular nitrogenase flux.
(EPS)

Figure S3 **Estimating ATP requirements from batch data.** Average growth rates and photon uptake fluxes from batch experiments were used to constrain the model. The maximum ATP hydrolysis flux (flux through the ATPM reaction) was calculated using these measurement constraints. The data points represent the calculated maximal ATP hydrolysis values for different batch experiments. The growth-associated ATP requirement (GAR, slope) and non-growth associated ATP requirement (NGAR, y-intercept) were estimated by linear regression of these data.
(EPS)

Table S1 **Reactions in the genome-scale metabolic network** ***i*** **Cce806.**
(XLSX)

Table S2 **Metabolites in the genome-scale metabolic network** ***i*** **Cce806.**
(XLSX)

Table S3 **Genome-scale metabolic network statistics for** ***i*** **Cce806.** Number of genes, reactions, metabolites, and network gaps in *i*Cce806.
(XLSX)

Table S4 **Effects of changing simulation conditions on ATP requirement parameters, GAR and NGAR.**
(XLSX)

Table S5 **Biomass compositions under ammonia-limited (AL), and light-limited (LL) chemostat conditions.**
(XLSX)

Table S6 **Experimental measurements for two chemostat conditions.**
(XLSX)

Table S7 **Transcriptomic data.** Fold change in mRNA expression levels between light-limited and ammonia-limited chemostats.
(XLSX)

Table S8 **Proteomic data.** Protein expression levels measured in light-limited and ammonia-limited chemostats.
(XLSX)

Text S1 **Supplemental methods for A) biomass calculations and B) batch growth rate calculations.**
(PDF)
