# Supplementary material for: Genome-Scale Modeling of Light-Driven Reductant Partitioning and Carbon Fluxes in Diazotrophic Unicellular Cyanobacterium Cyanothece sp. ATCC 51142
Source: PLoS Comput Biol. 2012 Apr 5;8(4):e1002460. doi: 10.1371/journal.pcbi.1002460 (PMC3329150; doi:10.1371/journal.pcbi.1002460)
Supplement: Text S1 — Supplemental methods for A) biomass calculations and B) batch growth rate calculations. (PDF) [file pcbi.1002460.s013.pdf]

A) Calculations for amino acid compositions and cyanophycin levels

The amino acid composition used in the protein synthesis equation (PROTSYN_CN) in our model and total number of amino acids (except Cys, Met, and Trp) in protein (P) and cyanophycin (C) were obtained by first solving an optimization problem that minimizes distance between relative amino acid composition in protein (x_p^i) and theoretical amino acid composition from genomic data ($x_p^{i,theoretical}$) excluding unmeasured amino acids (Cys, Met, and Trp).

$$
\begin{aligned}
\min \quad & \sum_{i \in AA \setminus \{Cys,Met,Trp\}} \left(x_P^i - x_P^{i,theoretical} \right)^2 \\
s.t \quad & \sum_{i \in AA \setminus \{Cys,Met,Trp\}} x_P^i = 1 \\
& P.x_P^i = M^i \, \forall i \in AA \setminus \{Cys,Met,Trp,Arg,Asn,Asp,Glu,G\ln\} \\
& P.x_P^{Arg} + C.x_C^{Arg} = M^{Arg} \\
& P.\left(x_P^{Asp} + x_P^{Asn} \right) + C.x_P^{Asp} = M^{Asn+Asp} \\
& P.\left(x_P^{Glu} + x_P^{G\ln} \right) = M^{Glu+G\ln} \\
& x_C^{Arg}, x_C^{Asp} = 0.5 \\
& x_P^i \ge 0
\end{aligned}
$$

In this method, the experimentally measured amino acid compositions (M^i, µmol/g AFDW) were used from our two chemostat experiments for light-limited (LL) and ammonia-limited (AL) conditions to constrain the values of P, C and x_p^i , such that the estimated amounts of each type of amino acid in protein and cyanophycin equaled their measured values. The theoretical fractions ($x_p^{i,theoretical}$) and measured values (M^i) used in the problem are listed in Table I below. Cyanophycin contains an equal amount of Asp and Arg, and therefore the fraction of these two amino acids in cyanophycin (x_C^i) is 0.5.

Table I. Experimental amino acid compositions in light-limited and ammonia limited chemostats.

Conditions	LL	AL	Theoretical		Molecular weight (g/mol)
Amino acids	M^i (umol/gDW)	M^i (umol/gDW)	Fraction from protein sequences	Adjusted fraction (exclude Cys, Met, Trp) $x_P^{i,theoretical}$	
Asp	450.507	219.702	0.050	0.053	132.098
Ser	122.154	116.098	0.063	0.066	105.096
Glu	298.283	291.201	0.066	0.069	146.124
Gly	177.042	181.462	0.065	0.068	75.07
His	27.968	31.493	0.019	0.020	155.162
Arg	307.891	108.699	0.045	0.047	175.22
Thr	115.397	120.574	0.057	0.060	119.122
Ala	214.011	201.620	0.066	0.069	89.096
Pro	106.474	104.816	0.046	0.048	115.132
Cys	NA	NA	0.010	Excluded	121.162
Tyr	75.157	66.697	0.033	0.035	181.188
Val	116.127	115.411	0.061	0.064	117.148
Met	NA	NA	0.020	0.000	149.214
Lys	96.985	102.871	0.055	0.058	147.2
Ile	98.619	98.032	0.076	0.079	131.174

**Leu**	164.729	165.450	0.111	0.116	131.174
**Phe**	73.953	71.227	0.041	0.043	165.188
**Trp**	NA	NA	0.014	Excluded	204.226
**Asn**	NA	NA	0.049	0.051	132.124
**Gln**	NA	NA	0.052	Excluded	146.15

It should be noted that the compositions for Cys, Met, and Trp are missing from the table as these amino acids were not stable enough to be measured and hence were excluded from the optimization. Consequently, the amount P estimated by the above method represents the number of amino acids except Cys, Met, and Trp contained in proteins. Additionally, Glu and Gln, as well as, Asn and Asp are measured as pooled metabolites, so the reported measured values in Table I for Asp and Glu are really $M^{Asn+Asp}$ and $M^{G\ln+Glu}$, respectively.

Solving the above problem, we obtained estimated amounts for P, C and mole fractions for all amino acids in protein, except Cys, Met and Trp. These mole fractions were then readjusted to account for these unmeasured amino acids. The mole fractions (y^i_{P*}) for all 20 amino acids in protein (P^*) were calculated using the three equations listed below, and the resulting values are reported in Table II.

$$y^i_{P^*} = \text{theoretical fraction based on protein sequences} \forall i \in \{Cys, Met, Trp\}$$

$$P^* = \frac{P}{1 - \sum_{i \in \{Cys, Met, Trp\}} y^i_{P^*}}$$

$$y^i_{P^*} = \frac{x^i_P \cdot P}{P^*} \forall i \in AA \setminus \{Cys, Met, Trp\}$$

Table II. Compositions and fractions of amino acids in protein

Amino acids	LL Conditon		AL Condition	
	x^i_P	y^i_{P*}	x^i_P	y^i_{P*}
**Asp**	0.059	0.056	0.054	0.052
**Ser**	0.061	0.058	0.059	0.056
**Glu**	0.080	0.076	0.080	0.076
**Gly**	0.088	0.084	0.092	0.088
**His**	0.014	0.013	0.016	0.015
**Arg**	0.046	0.044	0.050	0.048
**Thr**	0.057	0.054	0.061	0.058
**Ala**	0.106	0.101	0.102	0.097
**Pro**	0.053	0.051	0.053	0.051
**Cys**	Excluded	0.010	Excluded	0.010
**Tyr**	0.037	0.035	0.034	0.032
**Val**	0.058	0.055	0.058	0.055
**Met**	Excluded	0.020	Excluded	0.020
**Lys**	0.048	0.046	0.052	0.050
**Ile**	0.049	0.047	0.050	0.048
**Leu**	0.082	0.078	0.084	0.080
**Phe**	0.037	0.035	0.036	0.034
**Trp**	Excluded	0.014	Excluded	0.014
**Asn**	0.058	0.055	0.053	0.051
**Gln**	0.067	0.064	0.066	0.063
***P* (umol AA /gDW)**	2014.285		1976.969	

***C* (umol AA /gDW)**	431.012	18.384
***P** (umol AA /gDW)**	2108.126	2069.071

The mass ratio of the total protein to cyanophycin (g protein/g cyanophycin) was calculated using P* and C and the weighted average molecular weight for amino acids in each macromolecule. This mass ratio was then used to calculate the protein and cyanophycin concentrations, provided measured total protein concentration (Table III). The concentration of soluble metabolites that are also part of the biomass equations were taken from [1] and [2]. Table S5 contains a more detailed description of the complete biomass equations used. The biomass was adjusted for each condition so that the total biomass added up to 1 g per g AFDW.

Table III. Biomass compositions in light-limited (LL) and ammonia-limited (AL) chemostats.

	**LL Condition**			**AL Condition**		
**Biomass components**	Raw data values (g/L)	Raw data + computed data (g/gAFDW)	Adjusted data (g/gAFDW)	Raw data values (g/L)	Raw data + computed data (g/gAFDW)	Adjusted data (g/gAFDW)
Protein	0.028	0.473	0.397	0.017	0.211	0.210
Carbohydrates	0.012	0.197	0.165	0.040	0.502	0.502
Lipids	0.010	0.161	0.135	0.011	0.142	0.142
RNA	0.011	0.181	0.152	0.008	0.097	0.097
DNA	0.002	0.040	0.034	0.003	0.033	0.033
Cyanophycin *	NA	0.116	0.097	NA	0.002	0.002
Chlorophyll **	0.0014	0.024	0.020	0.001076	0.014	0.014
Ash-free dry weight (g AFDW/L)	0.059			0.079		
Dry weight (gDW)	0.179			0.1814		
Total		1.191	1.000		1.000	1.000

. Cyanophycin concentration was not measured experimentally, but computed using macromolecular protein concentration measurements, and mass ratio of total protein (P) to cyanophycin (C) obtained from solving the above optimization problem.
**. Chlorophyll concentration was measured under both chemostat condition, following methods described by Meeks et al. [3]. Since the total composition of biomass components in LL condition is not equal to 1, we rescaled the data so that the total fraction equals 1.

B) Batch growth simulations (supplement for Table 1)

For every batch in which the light intensities at 630nm and 680nm were varied, instantaneously measured growth rate and photon uptake rates were obtained. For each batch, we calculated the average and standard deviation for growth and photon uptake rates over the first 5 hour of the exponential growth phase, during which, the changes in growth and photon uptake rates were relatively constant (pseudo-steady state assumption). In addition, the predicted growth rate and its uncertainty were obtained by constraining the photon uptake fluxes to the average and the average ± standard deviation of the photon uptake fluxes respectively, while maximizing growth rate.

References

1. Harvey RJ, Dev IK (1975) Regulation in the folate pathway of Escherichia coli. Adv Enzyme Regul 13: 99-124.
2. Bennett BD, Kimball EH, Gao M, Osterhout R, Van Dien SJ, et al. (2009) Absolute metabolite concentrations and implied enzyme active site occupancy in Escherichia coli. Nat Chem Biol 5: 593-599.
3. Meeks JC, Castenholz RW (1971) Growth and photosynthesis in an extreme thermophile, Synechococcus lividus (Cyanophyta). Arch Mikrobiol 78: 25-41.
